# Dysregulated lipid metabolism in lymphangioleiomyomatosis pathogenesis as a paradigm of chronic lung diseases

**DOI:** 10.3389/fmed.2023.1124008

**Published:** 2023-01-19

**Authors:** Clara Bernardelli, Anna Caretti, Elena Lesma

**Affiliations:** ^1^Laboratory of Pharmacology, Department of Health Sciences, Università degli Studi di Milano, Milan, Italy; ^2^Laboratory of Biochemistry and Molecular Biology, Department of Health Sciences, Università degli Studi di Milano, Milan, Italy

**Keywords:** chronic lung diseases, inflammation, senescence-mediated inflammation, mTOR, lymphangioleiomyomatosis, lipids

## Abstract

A chronic inflammatory condition characterizes various lung diseases. Interestingly, a great contribution to inflammation is made by altered lipids metabolism, that can be caused by the deregulation of the mammalian target of rapamycin complex-1 (mTORC1) activity. There is evidence that one of mTOR downstream effectors, the sterol regulatory element-binding protein (SREBP), regulates the transcription of enzymes involved in the *de novo* fatty acid synthesis. Given its central role in cell metabolism, mTOR is involved in several biological processes. Among those, mTOR is a driver of senescence, a process that might contribute to the establishment of chronic lung disease because the characteristic irreversible inhibition of cell proliferation, associated to the acquisition of a pro-inflammatory senescence-associated secretory phenotype (SASP) supports the loss of lung parenchyma. The deregulation of mTORC1 is a hallmark of lymphangioleiomyomatosis (LAM), a rare pulmonary disease predominantly affecting women which causes cystic remodeling of the lung and progressive loss of lung function. LAM cells have senescent features and secrete SASP components, such as growth factors and pro-inflammatory molecules, like cancer cells. Using LAM as a paradigm of chronic and metastatic lung disease, here we review the published data that point out the role of dysregulated lipid metabolism in LAM pathogenesis. We will discuss lipids’ role in the development and progression of the disease, to hypothesize novel LAM biomarkers and to propose the pharmacological regulation of lipids metabolism as an innovative approach for the treatment of the disease.

## Introduction

Lungs rely on a unique lipid biology that ensures respiratory function and is involved in the regulation of the immune response. The alveolar area sustains active lipid metabolism to maintain surfactant homeostasis thus ensuring optimal respiration cycle. Mostly composed of phospholipids with 5–10% of cholesterol and small amount of sphingolipids, surfactant is essential in reducing the surface tension in the alveolar walls (1). Sterol-regulatory element-binding proteins (SREBPs) regulate lung lipid biosynthesis and sterol homeostasis at transcriptional level (2). This family of transcription factors promotes the expression of cholesterogenic and lipogenic genes involved in fat storage (3). Growing evidence shows that SREBPs are involved in numerous pathogenic processes such as endoplasmic reticulum stress and inflammation. LPS-challenged macrophages from mice with a targeted deficiency in the gene encoding SREBP-1a, failed to activate lipogenesis, secretion of IL-1β and gene encoding Nlrp1a, which is a core inflammasome component (4). Oishi et al. demonstrated that in macrophages, SREBP1 reprograms lipid metabolism to produce the anti-inflammatory polyunsaturated fatty acids thus promoting the late resolution of TLR4-induced gene activation (5). SREBP1 induction in alveolar type 2 cells of *Insig1/2*^Δ/Δ^ mice causes neutral lipid accumulation in type II cells and in the alveoli, resulting in pulmonary inflammation and airspace remodeling (6). Transcriptome network analysis reveals that SREBP1 activation promotes lipotoxicity that in turn induces inflammation and fibrosis in the lung (7). Recent data have shown that SREBP2 integrates cholesterol metabolism with NLRP3 inflammasome activation in macrophages (8). SREBP1 is regulated by the mammalian target of rapamycin (mTOR) (9) that is a regulator of lipid and nucleotide synthesis. Once activated, SREBP1 translocates from the endoplasmic reticulum to the nucleus to promote the transcription of the enzymes responsible for glugoneogenesis and lipogenesis (10).

Lymphangioleiomyomatosis (LAM) is a rare progressive lung disease characterized by lung cystic destruction, and the progressive loss of lung function (11). LAM occurs sporadically or associated to Tuberous Sclerosis Complex (TSC) (12). Loss of *TSC* gene function causes to dysregulated mTOR signaling (13). mTOR has a central role in cell metabolism, integrating environmental signals to promote anabolic processes (14). Recently, likely because of mTOR dysregulation, several studies indicated the alteration of lipid metabolism in LAM as an interesting target to uncover novel therapeutic approaches (15).

The minireview describes the current state of knowledge of emerging role of lipids in LAM considering their contribution in inflammation and in the pathogenesis and progression of chronic lung diseases.

## Lipids in pulmonary diseases

### Inflammation

Lung inflammatory diseases are tightly linked with aberrant SREBP activity in respiratory epithelial cells and with dyslipidemia. SREBP2-mediated lipid metabolism promotion correlates with (COVID-19)-induced cytokine storm (16). The increased expression of SREBP-1 after respiratory virus infection (i.e., SARS-CoV), regulates the accumulation of lipid droplets, cell organelles involved in the amplification of inflammatory mediators’ production (17). Dysregulation of free fatty acids, cholesterol, and ceramides homeostasis in the lungs is associated with the pathogenesis and the progression of chronic obstructive pulmonary disease (COPD). Cigarette smoke–induced alveolar accumulation of lipids is an important event that triggers inflammation in alveolar macrophages (18). Several studies have demonstrated that ceramides, the central hub of sphingolipids metabolism, are elevated in COPD and contribute to chronic inflammation (19). Idiopathic pulmonary fibrosis (IPF) is a progressive lung disease characterized by inflammation and fibrosis. As recently reviewed by Suryadevara et al. (20), bioactive lipid mediators derived from fatty acids, glycerolipids, phospholipids, and sphingolipids exhibit pro- or anti-fibrotic/inflammatory effects in lung tissues of IPF patients. Acute Respiratory Distress Syndrome (ARDS) is a severe form of clinical acute lung injury (ALI), both sharing severe inflammatory conditions. Phospholipase A2 enzymes (sPLA2s), that generate free fatty acids and lysophospholipids from glycerophospholipids, are upregulated in different cell types in the ALI/ARDS lung compartment. Significant evidence demonstrates that in these pathological settings, PLA2s are functionally important as regulators of inflammatory signaling (21). Caused by several factors, pneumonia is the inflammation localized to the terminal airways, the alveoli, and the interstitium of the lungs. A pilot study conducted in 2017 during community-acquired pneumonia (22), reported that monounsaturated and polyunsaturated fatty acid accumulation elicits an inflammatory response during pneumonia progression. Cystic Fibrosis (CF) is an inherited recessive disease. CF is caused by mutations in the CF transmembrane conductance regulator gene, with most of the mortality given by the lung dysfunction (23), being inflammation an independent risk factor for disease progression. We previously demonstrated that CF bronchial epithelial cells accumulate the sphingolipid ceramide (24), which contributes to CF airways inflammation (25), as well as glycerophospholipids and lyso-glycerophospholipids, the latest considered pro-inflammatory molecules (26), cholesterols, cholesterol esters and triacylglycerols (27), compared to normal cells.

### Senescence-mediated inflammation

Senescence is a stress-response process characterized by the irreversible arrest of the cell cycle following DNA damage. Senescent cells develop a senescence-associated secretory phenotype (SASP) to maintain their status and to communicate within their microenvironment. SASP factors, both released as soluble molecules or within extracellular vesicles (EVs), have strong biological activities on neighboring cells, including the promotion of inflammation. Indeed, the study of SASP in all *in vitro* generated models demonstrated the presence of the proinflammatory interleukin-6 (IL-6), CXC chemokine ligand 8 (IL-8), and monocyte chemoattractant protein 1 (28). Even if senescence allows the maintaining of tissue homeostasis, an irreversible proliferation arrest might limit the regenerative capacity of the tissue, and the presence of a pro-inflammatory milieu might sustain the onset of pathological condition and age-related disorders. In pulmonary diseases, accelerated or premature aging emerged to be significant. The oxidative stress caused by cigarette smoke induces senescence in alveolar epithelial cells (AEC) and endothelial cells that accumulate in COPD lungs, while in IPF the shortening of the telomeres might be responsible for the senescent status of fibroblasts and AEC (29). Of note, AEC might be driven to senescence also after the serine/threonine kinase mTOR hyperactivation (30). The constitutive activation of mTOR in the lung epithelial cells of transgenic mice results in weakness tight junctions’ (TJ), that is a sign of injury in the lung epithelium. In parallel, also epithelial to mesenchymal transition (EMT) was enhanced, ultimately contributing to lung fibrosis. In this study, however, senescence was not observed, but the authors suggested that this might be due to differences in the timing of mTOR activation compared to previous studies (31). Moreover, the peculiar contribution of the AEC to local inflammation might be ascribed also to the release of EVs. Interestingly, it was recently demonstrated that AEC released EVs that differs on their miRNA content, depending on the apical or basolateral origin site of their release. EVs released from the apical side of epithelial cells are enriched in miRNAs enhancing the mTOR signaling pathways, probably being responsible for the TJ vulnerability and for the EMT, which can cause lung injury (32). Of note, senescence and lipid metabolism share many regulatory proteins, among which mTOR has a crucial role (33). Senescent cells have increased lipids uptake and accumulation in lipids droplets (34) and it has recently arisen that the regulation of specific lipid species plays a critical role in senescence contributing to the chronic inflammation associated with SASP (35). For example, the prostaglandin E2 (PGE_2_), which is synthesized from the arachidonic acid, is expressed at higher levels in COPD fibroblasts compared to healthy controls and is responsible for the autocrine and paracrine spreading of senescence in neighboring cells, as well as of the induction of inflammatory response through the up-regulation of its receptors EP2 and EP4 in a cyclooxygenase 2 -dependent reactive oxygen species response (36). Notably, PGE_2_ is released also by epithelial cells to relax airways smooth muscle cells, indicating that the roles of lipid molecules in the crosstalk between cells in the lung environment is complex and needs further investigation (37). Additionally, the signaling lipid leukotrienes, that are SASP components, are secreted by senescent fibroblasts and contribute to worsen IPF promoting fibrosis in lung (38). Finally, lysophosphatidylcholines (LPCs) have SASP activity, being expressed at high levels in senescent fibroblasts and being capable to induce the secretion of IL-8 and IL-6 (35).

## LAM and mTOR

Lymphangioleiomyomatosis (LAM) primarily affects women of childbearing age. LAM cells are smooth muscle-like cells bearing a mutation in *TSC1* or *TSC2* tumor suppressor genes that causes the hyperactivation of Rheb with an increase of mTORC1 and adenosine 5′-monophosphate activated protein kinase (AMPK) activity (39, 40). AMPK control the energy levels by promoting the catabolic processes and by inhibiting the anabolic activities. Interestingly, tuberin-null cells show the AMPK hyperactivation which correlates with the cytoplasmic localization of p27Kip1 that negatively regulates the cyclin dependent kinase 2 (41). mTORC1 or mTORC2 induce the expression and the proteolytic process of SREBP1; therefore, mTORC1 hyperactivation, through SREBP1 and acetyl-CoA carboxylase, controls the lipid accumulation (10). Moreover, the synthesis of PC is controlled by mTORC1 through SREBP1 (9). As a consequence of the *TSC* mutations, the loss of heterozygosity in *TSC1* or *TSC2*, which causes the lack of hamartin or tuberin, leads to the hyperactivation of mTOR that promotes cell growth and proliferation by stimulating anabolic metabolism with the increase of protein and lipid synthesis (42, 43). Furthermore, constitutive mTOR activation leads to an increased invasion of lymphatics and lungs where LAM cells and wild type stromal cells form cysts with the consequent pneumothorax and progressive loss of pulmonary function (44).

The proliferation of LAM cells in the lung parenchyma is associated with diffuse cystic lesions and their infiltration into the lymphatic walls causes lymphatic abnormalities leading to damage or obstruction of lymphatic vessels (45). Lymphangiogenesis plays a pathogenetic role whereby LAM cells invade and spread through the lymphatic, a process that is related to a high levels of vascular endothelial growth factor (VEGF)-D (46). As well, nearby and into the LAM lesions, the increased expression of VEGF-A stimulates angiogenesis that also might be a mechanism by which LAM cells invade the circulation (47). Clinical manifestations of LAM include progressive dyspnea, cough, chest pain, recurrent pneumothoraces, and chylous complications including chylothorax, chyloptysis, and chylous ascites (13). Angiomyolipomas, benign tumors composed of LAM cells, smooth muscle cells, adipose tissue, and vessels, can often occur in LAM patients.

The discovery of the genetic basis of LAM cells led to consider the inhibition of mTOR hyperactivation as a therapeutic approach. Rapamycin indeed inhibits LAM cell proliferation resulting in the stabilization of the lung function, in the reduction of lymphatic abnormalities, and of the angiomyolipoma volumes, with no efficacy in the regression of the existing pulmonary lesions. So far, the treatment with rapamycin is the only approved for LAM (48).

After being classified as an interstitial lung disease, LAM has been recently reconsidered as a low-grade, destructive, metastasizing neoplasm for several features as the invasive LAM cell properties, the recurrency in donor allografts of patients who have undergone lung transplantation, and the metabolic reprogramming. However, LAM differs from other neoplasms for the bilateral and symmetrical disruption of the lung without a clear primary tumor site and dominant mass lesions (49).

We recently demonstrated that LAM/TSC cells, derived by chylous thorax of a LAM/TSC patient, have senescent features dependent from mTOR hyperactivation and the capability to induce senescence in neighboring cells (50). As demonstrated in other respiratory disease such as IPF, senescence might drive the progressive loss of parenchymal structure in LAM which ultimately causes the impairment of lung function (51). It has been demonstrated that LAM cells secrete molecules known to be SASP components comprised metalloproteinases (52), proinflammatory cytokines IL-6 (53) and IL-8 (50), kathepsin K (54), and VEGF-D (39) reinforcing the hypothesis of a LAM cell communication with the microenvironment and a SASP modulation of the remodeling of the lung parenchyma. Interestingly, IL-8, a potent neutrophil chemotactic factor that triggers chemotaxis and neutrophil activation through a phosphorylation cascade in the inflammatory response (55), has been demonstrated to be involved in the pathogenesis and progression of lung diseases such as ARDS and SARS CoV-2, suggesting the possibility to use IL-8 as a biomarker or as a therapeutic target (56). In LAM, besides its role in senescence, IL-8 might reinforce the senescence/inflammatory milieu driving the progression of the disease.

Furthermore, mTOR role in LAM senescence can be supported by the senolitic effect of rapamycin and the relapse following the suspension of rapamycin treatment caused by an irreversible senescent state, called geroconversion, sustained by mTOR hyperactivation (57).

## Lipids as novel potential therapeutic targets and biomarkers

### Pulmonary diseases

In view of the deep interplay between dysregulated lipid metabolism and chronic inflammatory condition that characterizes several lung diseases, restoring lipid homeostasis could represent a novel therapeutic approach. Herein, there are examples of preclinical studies focusing on the potential anti-inflammatory efficacy of compounds targeting lipid pathway in pulmonary diseases (Table 1). Treatment with A922500, a pharmacological inhibitor of acyl-CoA:diacylglycerol acyltransferase-1, inhibits lipid droplets biogenesis triggered by SARS-CoV-2 infection in A549 human epithelial cells and in primary human monocytes. Thereon, the synthesis of pro-inflammatory lipid production and cytokinesis is downregulated (58). Interestingly, we were able to also reduce glycerol- and cholesterol-based lipids, to promote fatty acids oxidation, to reduce inflammation in *in vitro* model of CF by reducing ceramide accumulation with Myriocin, the inhibitor of the rate-limiting step in sphingolipid biosynthesis (27). Finally, in an *in vivo* model, the bleomycin-induced lung fibrosis and inflammation in rats was attenuated by AK106-001616 that inhibits the cytosolic PLA2 responsible for the generation of pro-inflammatory eicosanoids (59).

**TABLE 1:** Anti-inflammatory efficient compounds targeting lipid pathways in pulmonary diseases.

Pulmonary disease	Lipid target	Anti-inflammatory compound	References
SARS-CoV-2 infection	Leukotrienes (LTB4 and cysLT)	A922500, DGAT-1 inhibitor	(61)
	Sphingosine	Fingolimod (FTY720), sphingosine analog	(74)
ALI/ARDS	Ceramide	D609, aSMAse inhibitor	(75)
	Sphingosine	Fingolimod (FTY720), Sphingosine analog	(76, 77)
CF	Ceramide	Myriocin, SPT inhibitor	(26, 78)
		Amitriptyline, Trimipramine, Desipramine, aSMAse inhibitors	(78)
	Glucosylceramide	Miglustat, GBA2 inhibitor	(80)
PF	Eicosanoids	AK106-001616, PLA2 inhibitor	(62)
	Cholesterol	Statins, HMG-CoA reductase inhibitors	(81)
Asthma	Cholesterol	Statins, HMG-CoA reductase inhibitors	(82)
	Diacylglicerols	R59949, DGKα inhibitor	(83)
COPD	Ceramide	Myriocin, SPT inhibitor	(84)
	Cholesterol	Atorvastatin, HMG-CoA reductase inhibitors	(85, 86)
LAM	Ganglioside D3	Rapamycin	(61)
	LPCs	Rapamycin Torin1	(64)
	AdPLA2 (PLA2G16)	MAFP	(66)
	DAG	Rintanserin ± chloroquine	(67)

cysLT, cysteinyl leukotriene; LTB4, leukotriene B4; DGAT, acyl-CoA:diacylglycerol acyltransferase-1; aSMAse, acid sphingomyelinase; SPT, serine palmitoyltransferase; PF, pulmonary fibrosis; GBA2, glucosylceramidase beta 2; PLA2, phospholipase A2; HMG-CoA, 3-hydroxy-3-methylglutaryl-coenzyme A; LPCs, lysophosphatidylcholines; MAFP, methyl arachidonyl fluorophosphate; AdPLA2, adipocyte phospholipase A2; DAG, diacylglycerol.

### LAM

Lymphangioleiomyomatosis (LAM) cells have a metabolic signature of increased fatty acid uptake and synthesis, a characteristic in common with cancer cells (60), making altered lipid species in LAM both disease relevant biomarkers and potential therapeutic targets.

For instance, in the perspective of a LAM immunotherapy, it was demonstrated that LAM lung sections of both patients and Tsc2^–/–^ mice have a strong positivity to the ganglioside D3 (GD3), whose expression is a characteristic that LAM cells maintain also after multiple passages *in vitro* (61). Interestingly, LAM patients have lower anti-GD3 antibodies in their serum compared to healthy controls and the *in vitro* treatment of LAM cells with commercial anti-GD3 antibodies plus human complement induce death in the 12–42% of the population. This might suggest an approach similar to melanoma, in which the targeting of GD3 by antibodies reduces tumor growth through the activation of the natural killer T cells (62).

Even if GD3 expression in LAM cells and lipids accumulation in the renal angiomyolipomas of LAM patients can be considered a consequence of mTOR deregulation (63), emerging researches demonstrate the existence of uncovered TSC1/2-dependent-mTORC1-independent regulation of lipids metabolism that may contribute to the LAM pathogenesis and progression.

In the first systematic study of the LAM lipidome, high levels of four LPCs (C16:0, C18:0, C18:1, and C20:4) were found in the plasma of women with LAM compared with healthy controls, suggesting novel potential biomarkers (64). LPCs are bioactive lipids generated by the activity of phospholipase A (PLA) on the precursor PC (9) and preclinical models of lung cancer showed that those molecules might have a direct role in tumor angiogenesis (65), a condition that, together with lymphangiogenesis, is characteristic also in pulmonary and extrapulmonary LAM (49). Interestingly, the increase of LPCs were also observed in Tsc2^–/–^ mouse embryonic fibroblasts (MEFs), consistently with the hypothesis that tuberin loss might enhance several phospholipid and neutral lipid species. The treatment with the mTOR inhibitors rapamycin or torin1, or the down-regulation of SREBP1 does not suppress LPCs, while the down-regulation of specific PLAs decreases selectively the proliferation of Tsc2^–/–^ MEFs compared with Tsc2^+/+^ MEFs (58). Among PLAs, the accumulation of the PLA2G16 isoform was observed in the lung nodules and in the renal angiomyolipomas of LAM patients, and it was demonstrated that the presence of tuberin negatively regulates PLA2G16 overexpression both *in vitro* and *in vivo*, affecting also the production of prostaglandins, that are critical mediators of chronic inflammation and cancer progression. In the same study, Tsc2^–/–^ MEFs treated with rapamycin or torin1 did not change the expression of PLA2G16, while the PLA inhibitor methyl arachidonyl fluorophosphonate (MAFP) reduced the growth and induced apoptosis on tuberin-deficient cells derived from LAM patient. Interestingly, the tuberin induced-expression in these cells prevents the effects of MAFP treatment (66).

Since mTORC1 hyperactivation causes the reduction of autophagy and induces a metabolic reprogramming of tuberin-deficient cells, a novel therapeutic approach was proposed in a recent study which demonstrates that Tsc2^–/–^ MEFs are sensitive to the treatment with rintaserin in combination with chloroquine, while the impact of those two drugs on Tsc2^+/+^ MEFs is minimal (67). Rintaserin is an inhibitor of the diacylglycerol kinase alpha (DGKA), that induces the formation of the phosphatidic acid from diacylglycerol, ultimately regulating the homeostasis of cell membranes (68). Tsc2^–/–^ MEFs increased the expression and the activity of DGKA, with a consequent 5-fold higher accumulation of phosphatidic acid compared to Tsc2^+/+^ MEFs and a higher capability to uptake nutrients from the extracellular space through macropinocytosis. The treatment with rintaserin blocks macropinocytosis and leads to the accumulation of diacylglycerol, reprogramming the phospholipid metabolism by reducing the storage of lipids in droplets and enhancing the synthesis of phospholipids (67). Interestingly, high levels of DGKA, DGKD, DGKQ, and DGKZ were found in the angiomyolipomas of TSC patients, that often develop LAM as pulmonary manifestation (69). *In vivo*, the injection of tuberin-deficient cells with the downregulation of DGKA does not cause the enlargement of lung alveoli observed in preclinical models of LAM (67, 70).

Taken together, these studies indicate that targeting of key metabolic pathways in lipids synthesis might be novel therapeutic approaches for LAM.

Moreover, lipids can be relevant biomarkers to understand LAM pathogenesis and progression. Indeed, the first evaluation of the LAM serum metabolome showed that there are differences in the metabolic profile when LAM patients were stratified according to menopausal status, lung function, disease burden and disease activity (15). These metabolic abnormalities involved almost exclusively sphingolipids, phospholipids and acylcarnitine fatty acids, reflecting metabolic processes downstream of mTOR. Remarkably, the observation that FEV_1_ loss, used as measure of airflow limitation, was related to sphingolipid and acylcarnitine fatty acid metabolism, might reflect the extent of lung parenchyma disruption, resulting in the loss of elastic recoil. In the same way, the airflow obstruction in patients with COPD is associated with glycerophospholipids and sphingolipids metabolites, suggesting a common mechanism involving mTOR hyperactivation (71). Moreover, uncovered changes in glycerophospholipids were observed in patients with sporadic LAM compared with LAM/TSC ones after rapamycin treatment (15).

Finally, the altered lipid metabolism in LAM cells can be exploited to identify novel metabolic imaging biomarkers for the disease. Indeed, the previously reported enhancing in PC levels and neutral lipids in LAM cells (64) allowed to test the *in vivo* uptake of [^18^F]fluorocholine (FCH) and [^18^F]fluoroacetate (FACE) to detect tuberin-deficient cells in solid tumors and to monitor the response to rapamycin through dynamic PET (72). [^18^F]FCH uptake, but not [^18^F]FACE uptake in tuberin-deficient xenografts was rapamycin-sensitive and *in vitro* study on tuberin-deficient cells indicated that this difference might be due to an accumulation of these two compounds in two different cells compartment. In fact, [^18^F]FCH is mainly incorporated into lipids, while [^18^F]FACE enters into mitochondria and can be used as marker of mitochondrial activity, which is not suppressed by rapamycin, confirming a specific metabolic feature of tuberin-deficiency in LAM cells (73).

## Conclusion

**FIGURE 1:**
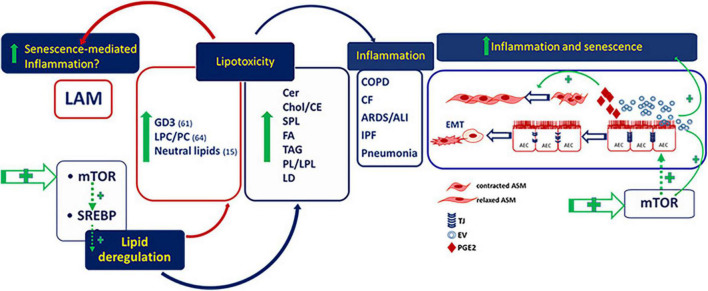
Deregulated lipids in lymphangioleiomyomatosis (LAM) and pulmonary diseases. GD3, ganglioside D3; LPC/PC, lysophosphatidylcholine/phosphatidylcholine; Cer, ceramide; Chol/CE, cholesterol/cholesterol esthers; SPL, sphingolipids; FA, fatty acids; TAG, riacylglycerols; PL/LPL, phospholipids/lysophospholipids; LD, lipid droplets; AEC, alveolar epithelial cells; ASM, airway smooth muscle.

Regulation of precise lipid species plays a critical role in senescence and senescence-mediated inflammation associated to chronic pulmonary diseases. Aberrant activity of SREBP and its upstream regulator, mTOR, is highly involved in lipotoxicity that induces inflammation and fibrosis in several lung diseases including LAM (Figure 1). The evidence of an altered lipid metabolism in tuberin-null cells, e.g., involving LPCs and GD3, and the demonstration of metabolic abnormalities in LAM patient serum, mainly LPCs, sphingolipids, phospholipids and acylcarnitine fatty acids, provides the need to promote the comprehension of lipid dysmetabolism in LAM as novel biomarkers for a metabolic signature for stratifying LAM patients and to suggest useful therapeutic targets, also to associate to mTOR inhibitor rapamycin, for the treatment of LAM.

## Author contributions

All authors listed have made a substantial, direct, and intellectual contribution to the work, and approved it for publication.
